# Associations of current and childhood socioeconomic status and health outcomes amongst patients with knee or hip osteoarthritis in a Mexico City family-practice setting

**DOI:** 10.1186/s12891-023-07107-0

**Published:** 2024-01-24

**Authors:** Julio Pisanty-Alatorre, Omar Yaxmehen Bello-Chavolla, Eduardo Vilchis-Chaparro, María Victoria Goycochea-Robles

**Affiliations:** 1 Instituto Mexicano del Seguro Social, Mexico City, Mexico; 2 Division of Research, Instituto Nacional de Geriatría, Mexico City, Mexico; 3 Cochrane-UNAM Group, Facultad de Medicina, Universidad Nacional Autónoma de México, Mexico City, Mexico

## Abstract

**Objectives:**

To examine the association of current and childhood socioeconomic status (SES) with patient-reported functional status, quality of life and disability in patients with knee or hip osteoarthritis (OA).

**Methods:**

Cross-sectional study amongst individuals seeking care for any medical reason in a primary care family-practice clinic in Mexico City. We included individuals with self-reported doctor-diagnosed arthritis, recruited through waiting-room posters and invitations by treating family physicians. We administered a survey using validated Spanish language versions of the Western Ontario and McMaster Universities Osteoarthritis Index (WOMAC), the Osteoarthritis of Lower Limbs and Quality of Life (AMICAL), and the Stanford Health Assessment Questionnaire-Disability Index (HAQ-DI). To estimate current and childhood SES, we collected data on education level and occupation type for both the patient and their parents, as well as using a validated tool to estimate income quintile.

**Results:**

We recruited 154 patients and excluded 8 patients. There was a high correlation between outcome scores. Estimated income and education levels were correlated with WOMAC, AMICAL and HAQ-DI scores, and significant differences were found in all scores by occupation type. The associations for current SES variables and outcome scores remained significant independently of age, sex, BMI, and presence of diabetes or hypertension, and were largely explained by current income in mutually adjusted models. Childhood SES – in particular as measured through maternal education – was best correlated with AMICAL scores, though its effect seemed largely mediated by its association with current SES.

**Conclusions:**

Current Socioeconomic Status impacts functional status, quality of life and disability amongst OA patients in Mexico City. The WOMAC, AMICAL and HAQ-DI scores correlate with each other and are all potentially useful markers of disease severity. More research is needed to elucidate the relationships between childhood SES and OA outcomes. Awareness of life-course SES may be useful in identifying patients at risk for worse outcomes.

## Introduction

Socioeconomic Status (SES) has attracted increasing interest as a determinant of both prevalence and outcomes for chronic health conditions. While observations relating socioeconomic status and health date at least as far back as the late 18th century [1], systematic collection of data in the past 50 years has greatly improved our understanding of the extent of health disparities [2–5]. The literature on health disparities regarding musculoskeletal disorders, such as osteoarthritis (OA), is less extensive than in other areas of medicine, but has garnered increased attention in the last few decades [6–9]. Various studies have examined the relationship between SES and OA prevalence, showing higher prevalence rates amongst lower-SES groups [10–17]. Similarly, a growing number of studies are beginning to paint a picture of a relationship between lower SES and worse OA outcomes [18–21].

Life-course epidemiology has identified lower SES during childhood as an important predictor of adult health [22]. A growing body of evidence shows an association between low childhood SES and increased prevalence of arthritis and of musculoskeletal pain (of which knee and hip OA probably play a large part). This relationship is partially mediated by adult SES, consistent with the pathway model of life-course epidemiology [23, 24]. To our knowledge, only one study has directly examined the relationship of childhood SES with arthritis outcomes. It found that low childhood SES is associated with higher disability and worse physical health amongst patients with arthritis (of whom most suffered from OA), after adjusting for current SES, age, sex, race, Body Mass Index (BMI) and comorbidities [20].

Since the relationship of life course SES and OA outcomes can be context-dependent, an important research gap remains in determining this relationship in Low and Middle Income countries and, in particular, in Latin America, where exposure to low SES throughout the life-course is higher [25].

The aim of this study was to examine the association of current and childhood SES with patient-reported functional status, quality of life and disability in a sample of patients with self-reported doctor-diagnosed primary knee or hip osteoarthritis seeking care in a family practice setting in Mexico City.

## Methods

### Study design and population

We conducted a cross-sectional study amongst individuals seeking care for any medical reason in a primary care family-practice clinic belonging to the Mexican Social Security Institute (IMSS) in Mexico City. We included adult patients 40 years or older with self-reported doctor-diagnosed knee or hip OA who signed informed consent. We excluded patients suffering from rheumatoid arthritis or other rheumatic conditions susceptible to causing secondary OA, as well as those who did not complete the general patient data questionnaire or withdrew consent after completion of questionnaires. There were no other exclusion criteria regarding sex, body size, or any other patient characteristic.

Patients were recruited through waiting room posters and invitations by family physicians from the clinic. At study entry, patients were asked the following question: “Has a physician ever told you that your knees or hips have osteoarthritis, osteoarthrosis, or that your joints are *worn out*?” Patients who answered positively were invited to complete the questionnaires. Because recruitment posters and invitations were targeted at patients with OA, all patients who approached the study answered affirmatively.

### Measurements

#### Outcome measurements

To assess functional status, we used the Western Ontario and McMaster Universities Osteoarthritis Index (WOMAC). The WOMAC is a widely used instrument to evaluate functional status for patients with osteoarthritis of the knee or hip. It consists of 24 questions about pain (3 questions), stiffness (2 questions) and physical function (17 questions). Each question is scored on a Likert scale from 0 to 4, leaving a total score ranging from 0 to 96, with higher scores indicating worse functional status [26, 27]. The Spanish-language version of the questionnaire has been validated [28].

To measure disease-specific quality of life we used the Osteoarthritis of Lower Limbs and Quality of Life (AMICAL) questionnaire. The questionnaire consists of 43 questions addressing quality-of-life domains such as physical activities, mental health, social functioning and social support. Each question is scored on a Likert scale from 0 to 10, and scores from 7 questions are reversed, leaving a total score ranging from 43 to 430 with higher scores indicating worse quality of life [29]. The questionnaire has been translated and culturally adapted into Spanish, and the version has been shown to have reliable test characteristics [30].

Finally, we used the Stanford Health Assessment Questionnaire-Disability Index (HAQ-DI) to assess degree of disability. The HAQ-DI consists of 20 questions concerning a patient’s ability to get dressed, get up, eat, walk, wash, reach for objects, grab things and carry out daily activities. Each question is scored on a scale ranging from 0 (no difficulty) to 3 (unable to do it). Additionally, patients are asked whether they need specialized equipment or assistance from others to carry out these activities. The highest score from each category is considered, and the 8 categories are averaged, leaving a score ranging from 0 to 3, with higher scores indicating more disability [31, 32]. The Spanish-language version has been validated [33].

#### SES measurements

We asked patients to complete a general data questionnaire including questions on level of education and occupation type for both themselves and their parents.

In Mexico, only about 20% of adults aged 50 or over have completed high-school, and there are significant inequities between groups achieving different levels of education. We therefore categorized level of education according to the highest achieved level out of 4 into which the school system is divided, leaving a total of 5 categories: less than elementary school (< 6 years), complete elementary school (6 years), middle-school (9 years), high-school (12 years), and college-level education (the equivalent to a B.A degree) or higher.

Occupation was measured as patients self-classifying their predominant lifetime occupation into one of four types: Manual labor, non-managerial office work, managerial work and “home occupation” (mostly women who identified as housewives). This last category is widely used in surveys in Mexico.

Direct questions about total annual income are notoriously unreliable in Mexico. Therefore, to measure income, we administered Gutiérrez et al.’s “Simplified measurement of socioeconomic status” questionnaire, which has been shown to reliably estimate income quintile in the general Mexican population. The questionnaire consists of 8 questions pertaining possession of goods and access to services, namely: owning a DVD player, a microwave oven, a phone, a home, a computer and a car; and having cable TV and internet service at home. Each good or service is multiplied by a weight – obtained from a national income survey – and then added together. Finally, the resulting index is classified into quintiles (Higher quintiles meaning greater income). The authors that developed this index have shown that it has a high correlation (Spearman’s rho of 0.85) with the income quintiles of the national income distribution as measured through much more extensive questionnaires in specialized surveys [34]. Because only 3% of patients in our sample had an estimated income in the lowest quintile, we grouped them with patients with estimated income in the second-lowest quintile.

Finally, childhood SES was assessed through the proxies of maternal and paternal education level, and maternal and paternal occupation type, which were categorized in the same manner as the participants’.

### Statistical analysis

We used descriptive statistics for all variables. In order to assess the similarity between the three assessed outcome scores, we calculated pairwise Pearson correlation coefficients. For ordinal variables education level, paternal and maternal education, and income quintile, we measured the correlation with the patient-reported outcomes by calculating Spearman coefficients (“rho”). For categorical variables occupation type, paternal and maternal occupation we used Kruskal-Wallis tests to identify differences in health outcomes, with post-hoc pairwise comparisons using Dunn tests with Bonferroni correction for comparisons against a specified group (Managerial office work).

In order to adjust for possible confounders (age and sex) and for the effect of mediators that we considered not of interest (BMI and presence of Diabetes and Hypertension), we fit linear regression models of all 3 outcomes against each current or childhood SES variables, first independently and then concurrently. We squared-root-transformed the WOMAC score in all models as this allowed fulfilling the necessary statistical assumptions for lineal models. The highest education and income categories were used as reference categories, while “Managerial office work” was used as reference category for variables pertaining to occupation.

To further analyze the relationships between childhood and adult SES and outcomes, we chose the variable for each life course stage that best predicted the outcomes overall which led us to fit models regressing outcomes against maternal education and current income quintile, adjusting for age, sex, BMI and presence of diabetes and hypertension. Finally, we dichotomized these two variables to construct “SES trajectories” out of the four possible resulting combinations (Low Maternal Education/Low Current Income; Low Maternal Education/ High Current Income; High Maternal Education/Low Current Income; and High Maternal Education/High Current Income), and regressed the outcomes against this new compound variable adjusting for covariates.

All statistical analyses were done using R software version 4.1.0, with a chosen significance threshold value of p < 0.05. Missing data were omitted from each analysis.

## Results

### Participants

**Fig. 1:**
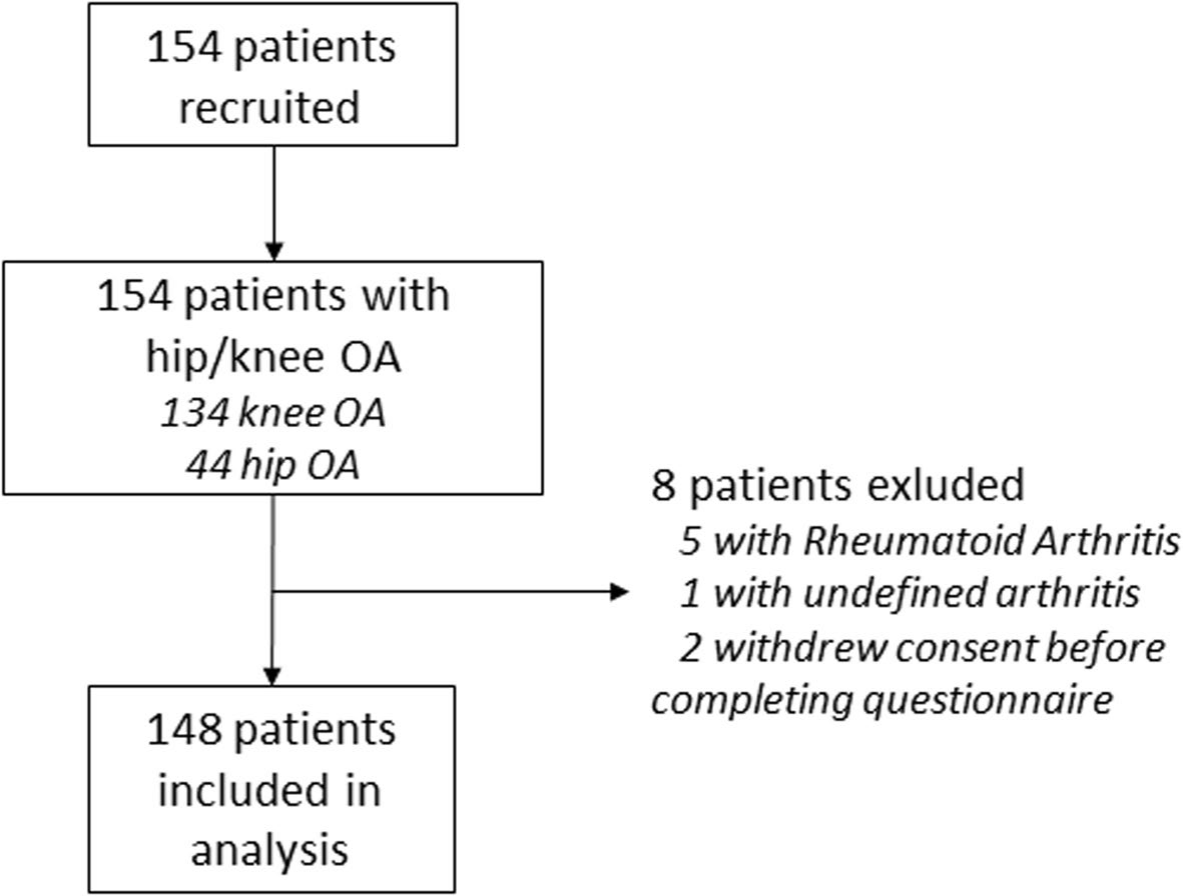
Study participants flowchart OA: Osteoarthritis

We recruited 154 patients and excluded 8 patients (5 with rheumatoid arthritis, 1 with undifferentiated arthritis and 2 who withdrew consent), leaving a total of 146 patients for final analysis (Fig. 1).

Relevant patient characteristics are shown in Table 1. A majority (80%) of patients were female, with a mean age of 69.4 years.

**Table 1:** Patient characteristics

	**Mean ± SD**	
**Age, years**	69.5 ± 10.2	
**Body Mass Index, kg/m** ^**2**^	28.4 ± 4.9	
**WOMAC Score** *n missing = 1*	44.6 ± 20.3	
**AMICAL Score** *n missing = 4*	235.6 ± 73.8	
**HAQ-DI Score** *n missing = 4*	1.14 ± 0.65	
	**n**	**%**
**Sex, female**	117	80
**Smoking history, pack years**		
0	93	64
>0, ≤ 5	26	18
>5, ≤ 10	8	5
>10, ≤ 15	2	1
≥15	17	**12**
**Diabetes**	41	28
**Hypertension**	76	52
**Education**		
Less than elementary (<6 years)	25	17
Elementary school (6 years)	29	20
Middle-school (9 years)	31	21
High-school (12 years)	39	27
College level or above	22	15
**Occupation type**		
Manual labor	67	47
Non-managerial office work	30	21
Managerial	22	15
Home	25	17
**Estimated income quintile**		
I or II	21	14
III	30	21
IV	31	21
V	64	44
**Paternal education**		
Less than elementary (<6 years)	48	36
Elementary school (6 years)	55	41
Middle-school (9 years)	12	9
High-school (9 years)	7	5
College level or above	13	9
**Maternal education**		
Less than elementary (<6 years)	66	45
Elementary school (6 years)	53	36
Middle-school (9 years)	7	5
High-school (9 years)	11	8
College level or above	4	3
**Paternal occupation**		
Manual labor	111	79
Non-managerial office work	10	7
Managerial	20	14
**Maternal occupation**		
Manual labor	36	25
Non-managerial office work	9	6
Managerial	4	3
Home	94	66

*SD* Standard Deviation; *kg/m*^2^ Kilograms per meter squared, *WOMAC* Western Ontario and MacMaster Universities Osteoarthritis Index, *AMICAL* Osteoarthritis of Lower Limbs and Quality of Life; *HAQ-DI* Health Assessment Questionnaire Disability Index

### Correlations between outcome variables

**Fig. 2:**
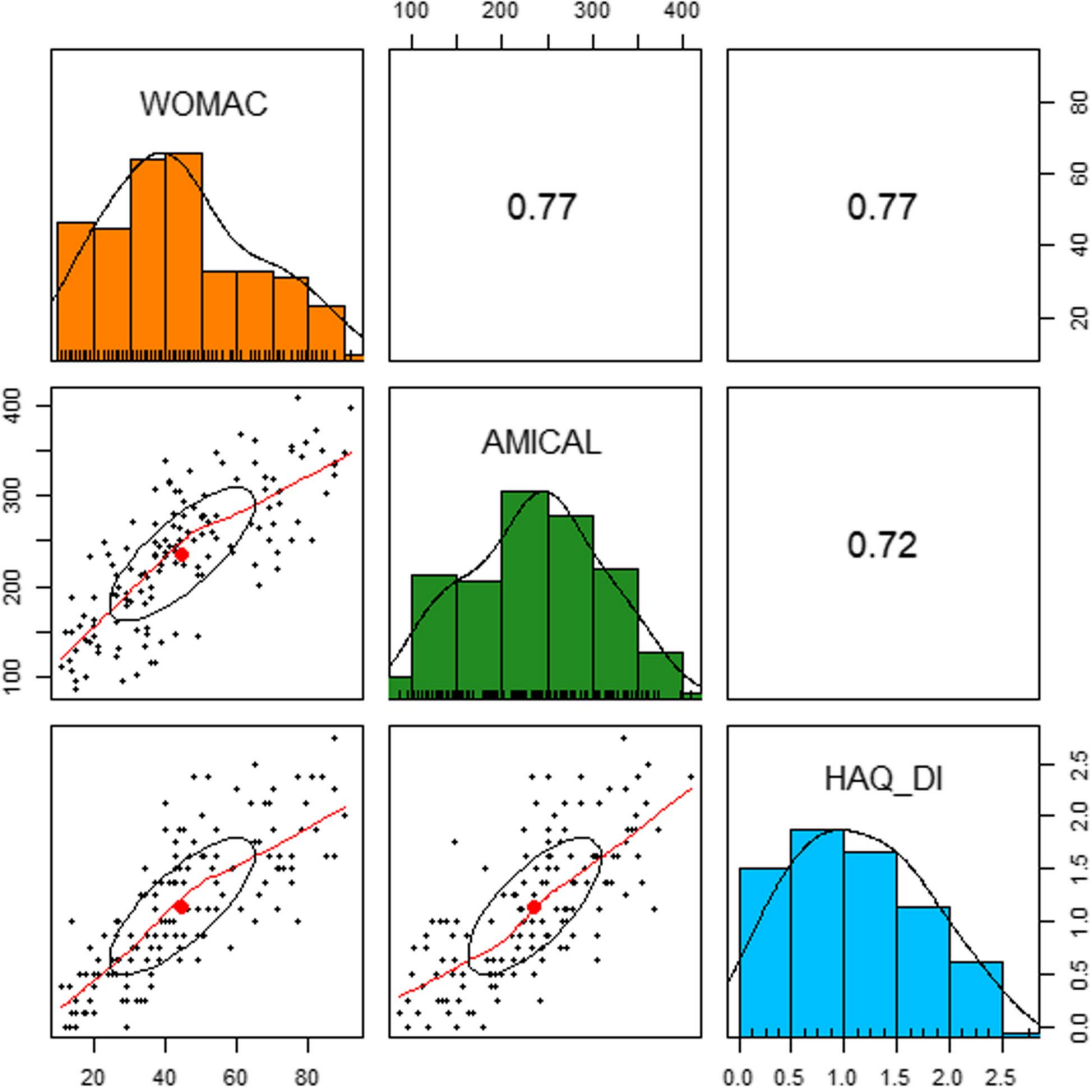
Correlations between outcome variables Pairwise Pearson correlation coefficients between WOMAC, AMICAL and HAQ-DI with associated histograms/density plots and correlation scatterplots

There was a highly significant and strong correlation between the WOMAC, AMICAL and HAQ-DI scores, with pairwise Pearson correlation coefficients > 0.7 (p < 0.001) for the three possible pairs of scores (Fig. 2).

### Current and childhood SES and outcomes

**Fig. 3:**
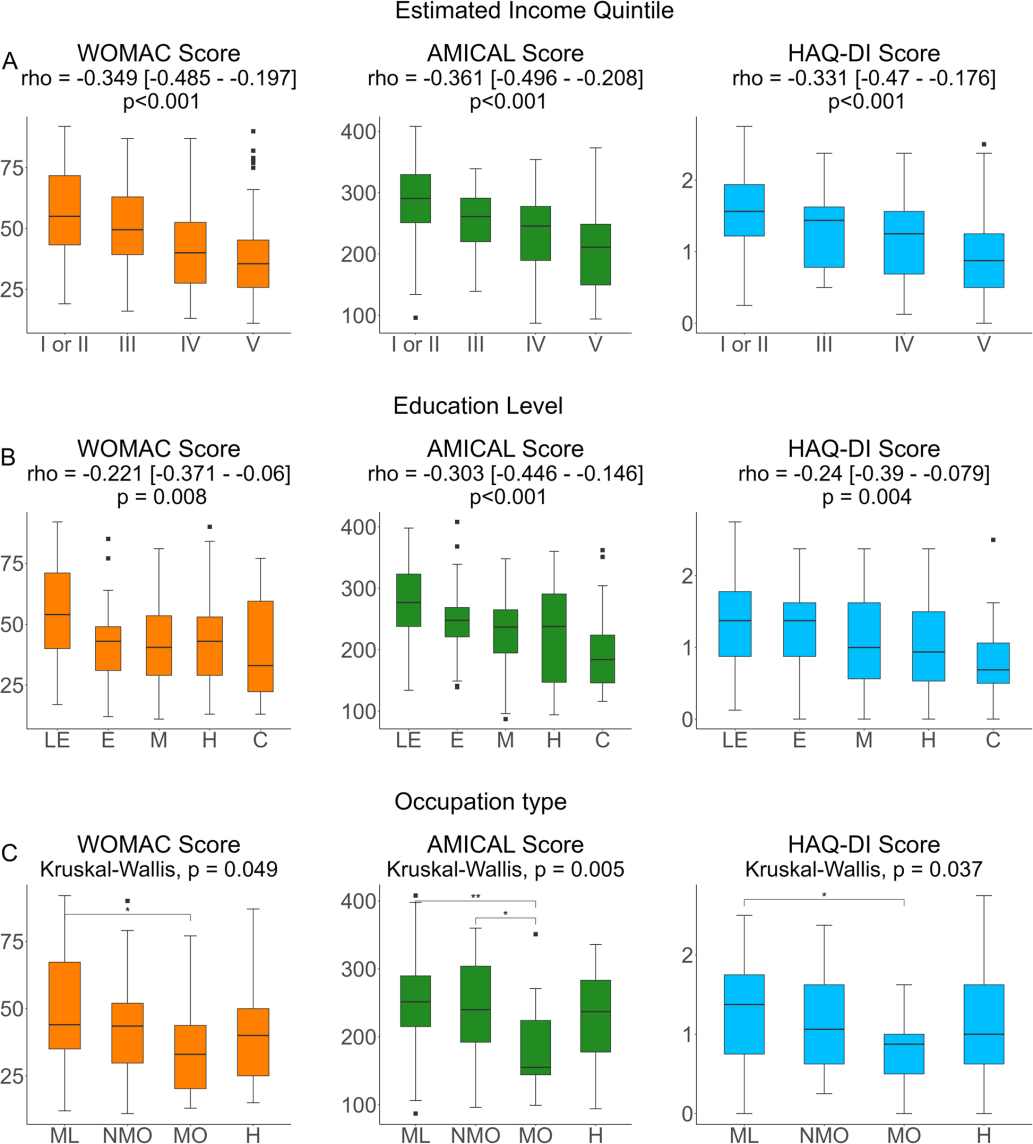
Outcome Scores by current Socioeconomic Status Categories Outcome scores by **A** *) *Current Estimated Income Quintile, **B** *)* Education Level & **C** *)* Occupation Type. Spearman analysis for trend was conducted for **A** and **B**. Kruskall-Wallis testing was conducted for **C**, with post-hoc Dunn tests with Bonferroni correction. *WOMAC*: Western Ontario and MacMaster Universities Osteoarthritis Index; *AMICAL*: Osteoarthritis of Knee and Hip and Quality of Life; *HAQ-DI*: Stanford Health Assessment Questionnaire Disability Index. For education level, *LE*: less than elementary (<6 yr), *E*: elementary (6 years), *M*: Middle-school (9 years), *H*: High-school (12 years), *C*: College-level or higher (>12 years). For occupation type, *H*: Home, *ML*: Manual Labor, *NMO*: Non-managerial office work, *MO*: Managerial office work. *Significant at the p<0.05 level; ** Significant at the p<0.01 level

Analysis of current SES variables showed that all three outcome scores were inversely correlated with both estimated current income quintile (Fig. 3A) and higher education level (Fig. 3B). In general, there were significant differences in all three outcome scores according to patients’ occupation. However, subgroup analysis showed that compared to patients with managerial office work, only patients dedicated to manual labor had significantly higher scores (Fig. 3C).

**Fig. 4:**
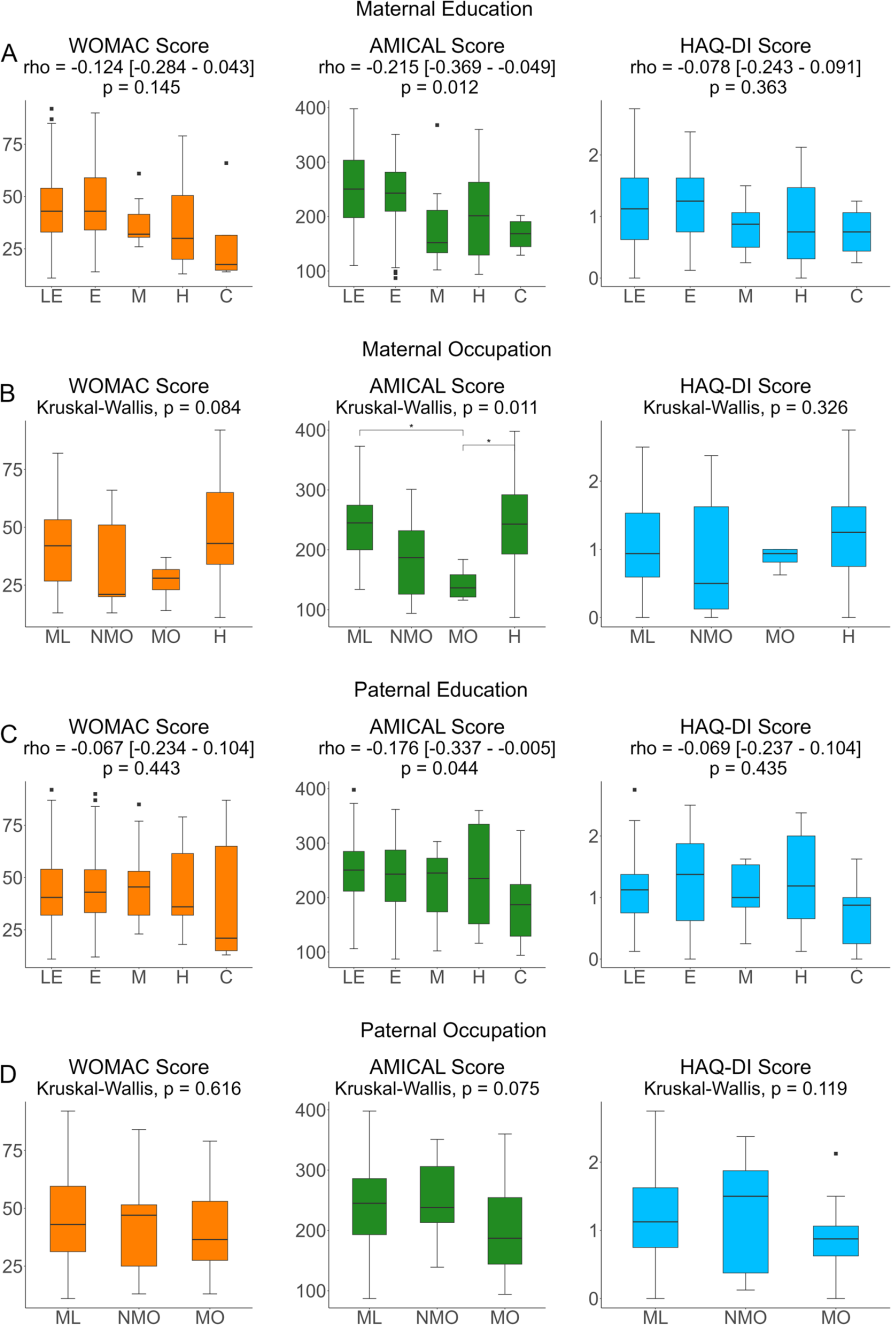
WOMAC, AMICAL and HAQ-DI Scores by childhood SES Outcome scores by **A**) Maternal Education **B**) Maternal Occupation type **C**) Paternal Education and **D**) Paternal Occupation type. Spearman analysis for trend was conducted for **A** and **C**. Kruskall-Wallis testing was conducted for **B **and **D**, with post-hoc Dunn tests with Bonferroni correction. *WOMAC*: Western Ontario and MacMaster Universities Osteoarthritis Index; *AMICAL*: Osteoarthritis of Knee and Hip and Quality of Life; *HAQ-DI*: Stanford Health Assessment Questionnaire Disability Index. For education level, *LE*: less than elementary (<6 yr), *E*: elementary (6 years), *M*: Middle-school (9 years), *H*: High-school (12 years), *C*: College-level or higher (>12 years). For occupation type, *H*: Home, *ML*: Manual Labor, *NMO*: Non-managerial office work, *MO*: Managerial office work. *Significant at the p<0.05 level; ** Significant at the p<0.01 level

Regarding childhood SES, both maternal and paternal education were inversely correlated with AMICAL scores, but not WOMAC or HAQ-DI scores. There were significant differences in AMICAL, but not WOMAC or HAQ-DI scores, by maternal occupation type, and no significant differences in any score by paternal occupation type (Fig. 4).

**Fig. 5:**
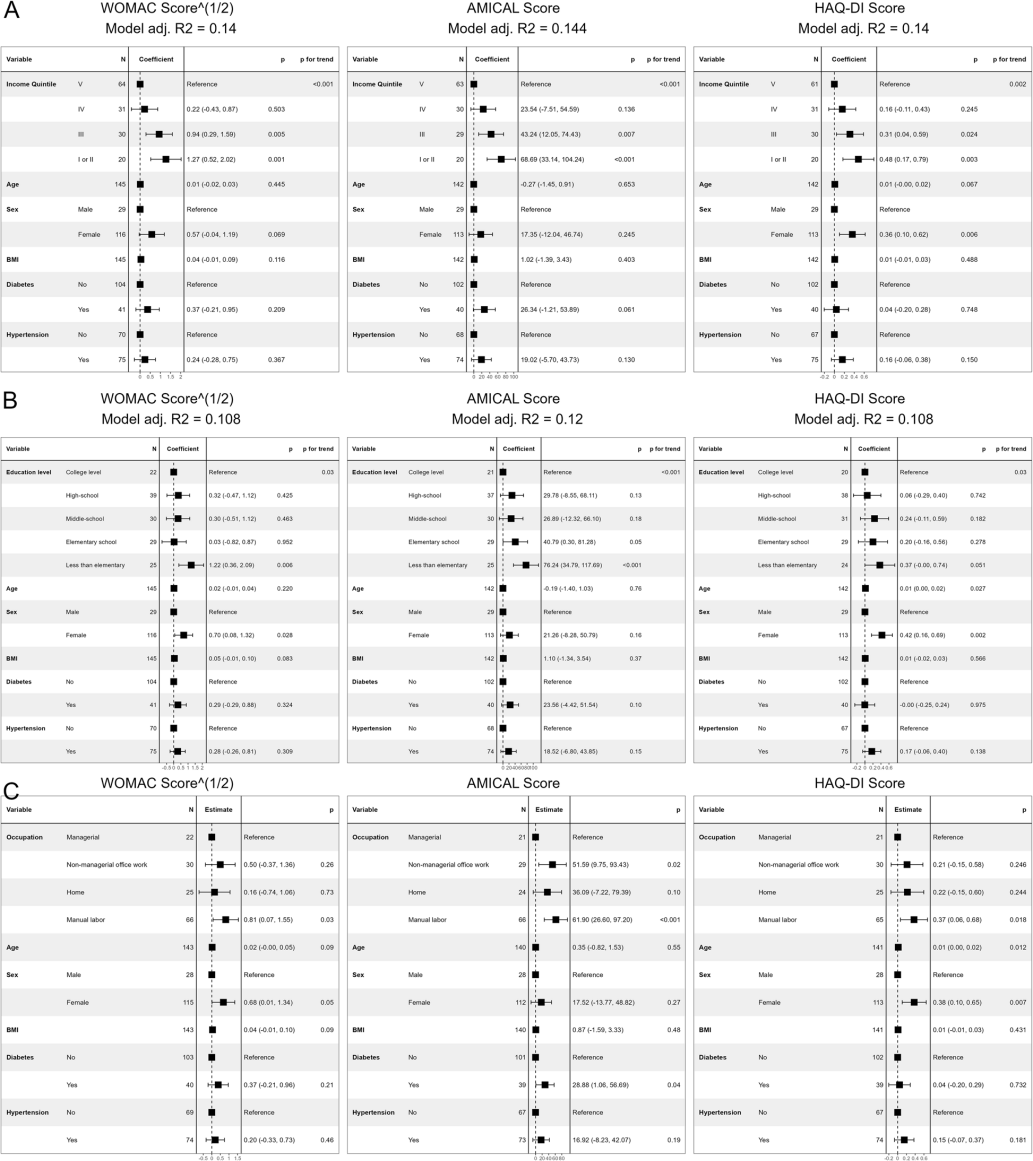
Linear regression models of effect of current Socioeconomic Status measures on outcome scores **A** Beta coefficients of regression models only including current income level. **B** Beta coefficients of regression models only including  education level. **C** Beta coefficients of regression models only including occupation type. *WOMAC*: Western Ontario and MacMaster Universities Osteoarthritis Index; *AMICAL*: Osteoarthritis of Knee and Hip and Quality of Life; *HAQ-DI*: Stanford Health Assessment Questionnaire Disability Index; *BMI*: Body Mass Index (kg/m^2^)

Linear regression models demonstrated that all three current SES measures (Current Income, Education Level and Occupation type) were associated with all three outcome scores (WOMAC, AMICAL and HAQ-DI) independently of age, sex, BMI, and presence of diabetes or hypertension, except for Occupation type with WOMAC scores which did not reach statistical significance (Fig. 5).

**Fig. 6:**
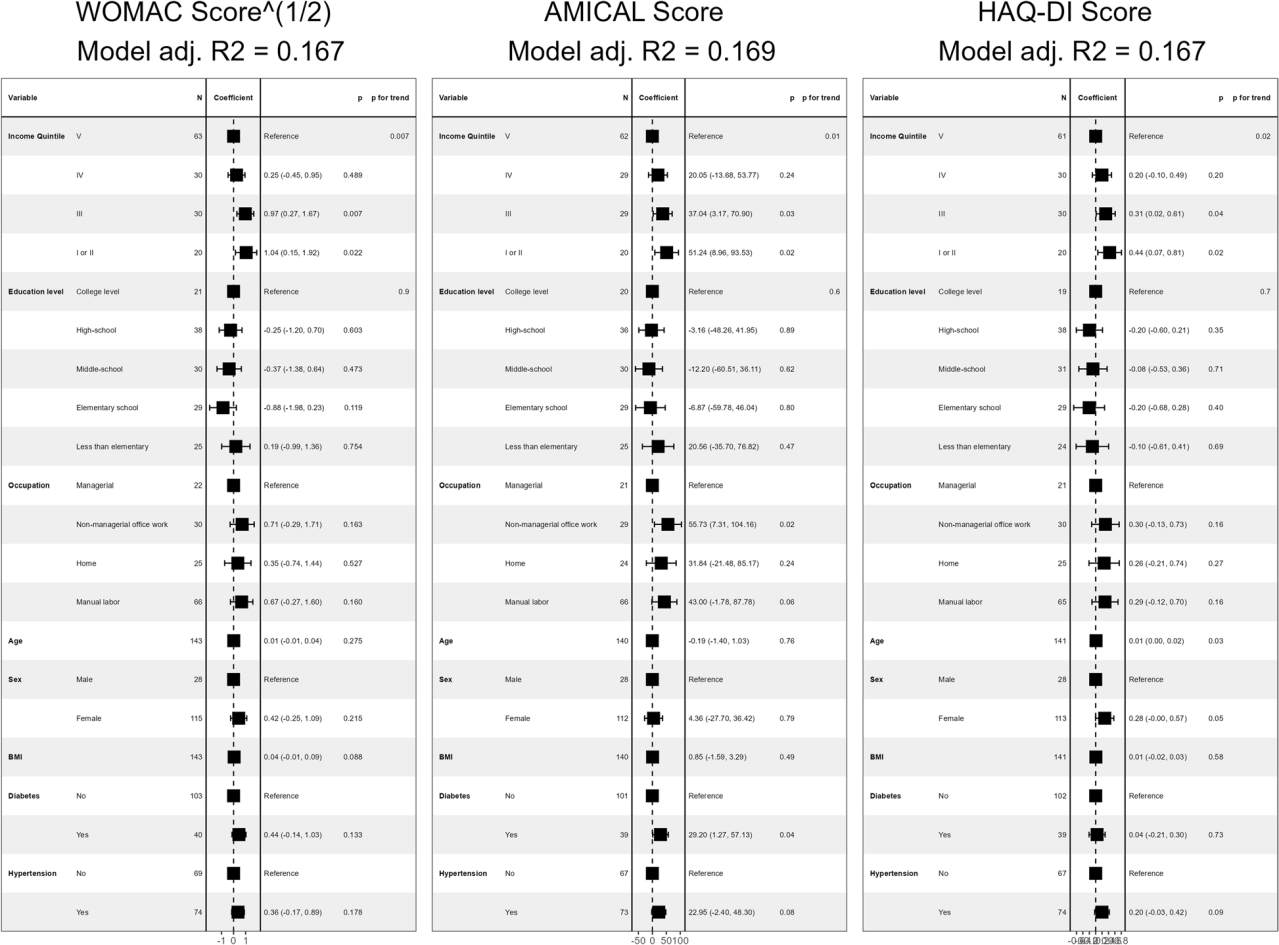
Concurrent effect of SES measures on outcome scores Beta coefficients of regression models including current income, education level and occupation type on outcome scores. *WOMAC*: Western Ontario and MacMaster Universities Osteoarthritis Index; *AMICAL*: Osteoarthritis of Knee and Hip and Quality of Life; *HAQ-DI*: Stanford Health Assessment Questionnaire Disability Index; *BMI*: Body Mass Index (kg/m^2^)

However, when all three current SES variables were entered concurrently in linear regression models, only current income quintile remained associated with the three outcome scores. Occupation type was associated only with the AMICAL score, while the association between education and any score disappeared (Fig. 6).

**Fig. 7:**
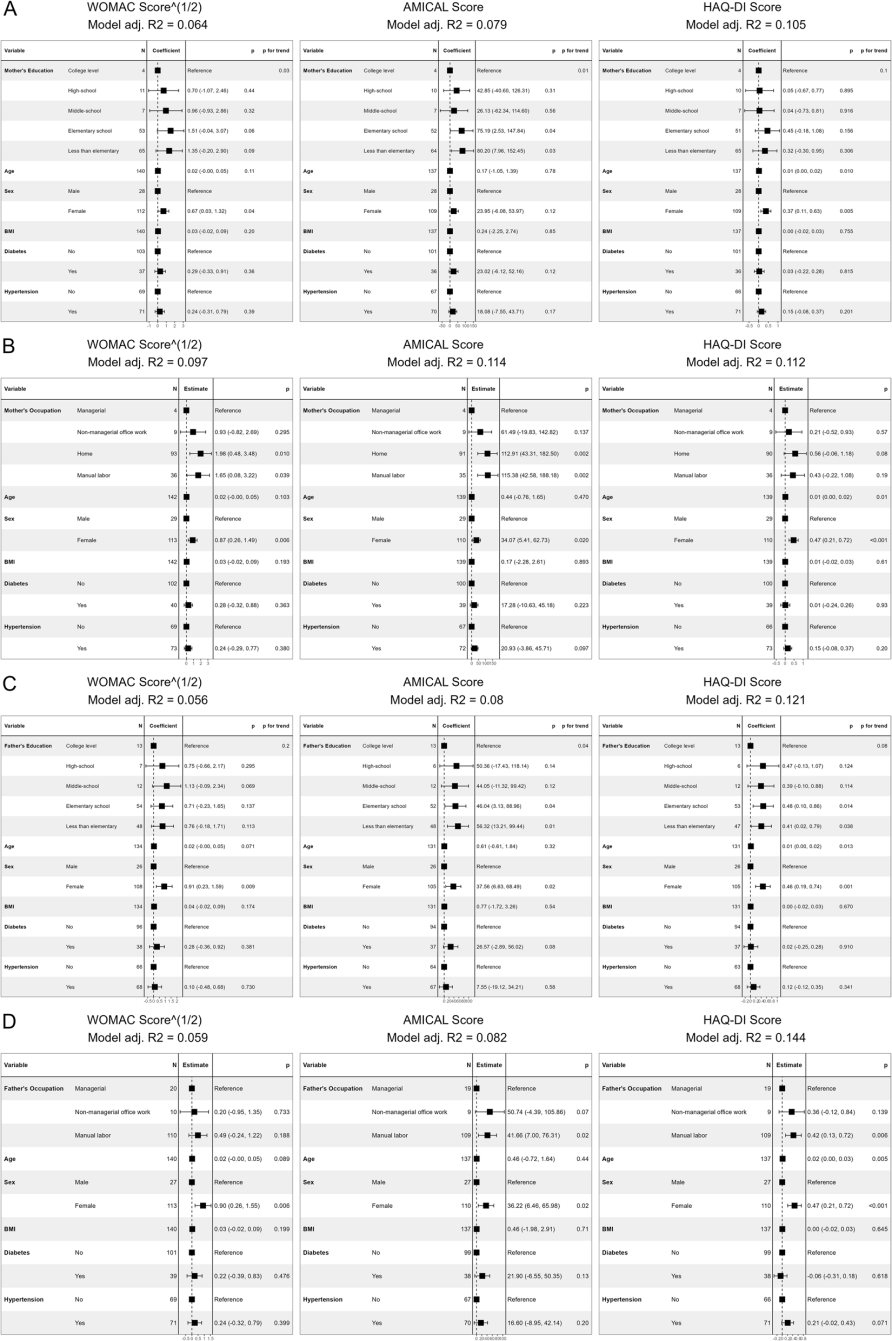
Linear regression models of effect of childhood Socioeconomic Status measures on outcome scores **A** Beta coefficients of regression models only including maternal education level. **B** Beta coefficients of regression models only including  maternal occupation type. **C** Beta coefficients of regression models only including paternal education level. **D** Beta coefficients of regression models only including paternal occupation type. *WOMAC*: Western Ontario and MacMaster Universities Osteoarthritis Index; *AMICAL*: Osteoarthritis of Knee and Hip and Quality of Life; *HAQ-DI*: Stanford Health Assessment Questionnaire Disability Index; *BMI*: Body Mass Index (kg/m^2^)

Similarly, after adjusting for age, sex, BMI and presence of Diabetes and hypertension, the association of maternal and paternal education with AMICAL scores remained significant. Additionally, maternal education was associated with the WOMAC, but not the HAQ-DI score. Paternal education was not associated with either the WOMAC or the HAQ-DI score. Similarly, maternal manual occupation was associated with significantly higher AMICAL and WOMAC scores, but not HAQ-DI scores, while paternal manual occupation was associated with the AMICAL and HAQ-DI scores (Fig. 7).

Strong collinearity between the different childhood SES variables (e.g. Spearman’s rho for maternal and paternal education = 0.78) prevented us from entering all childhood SES variables in a single model.

**Fig. 8:**
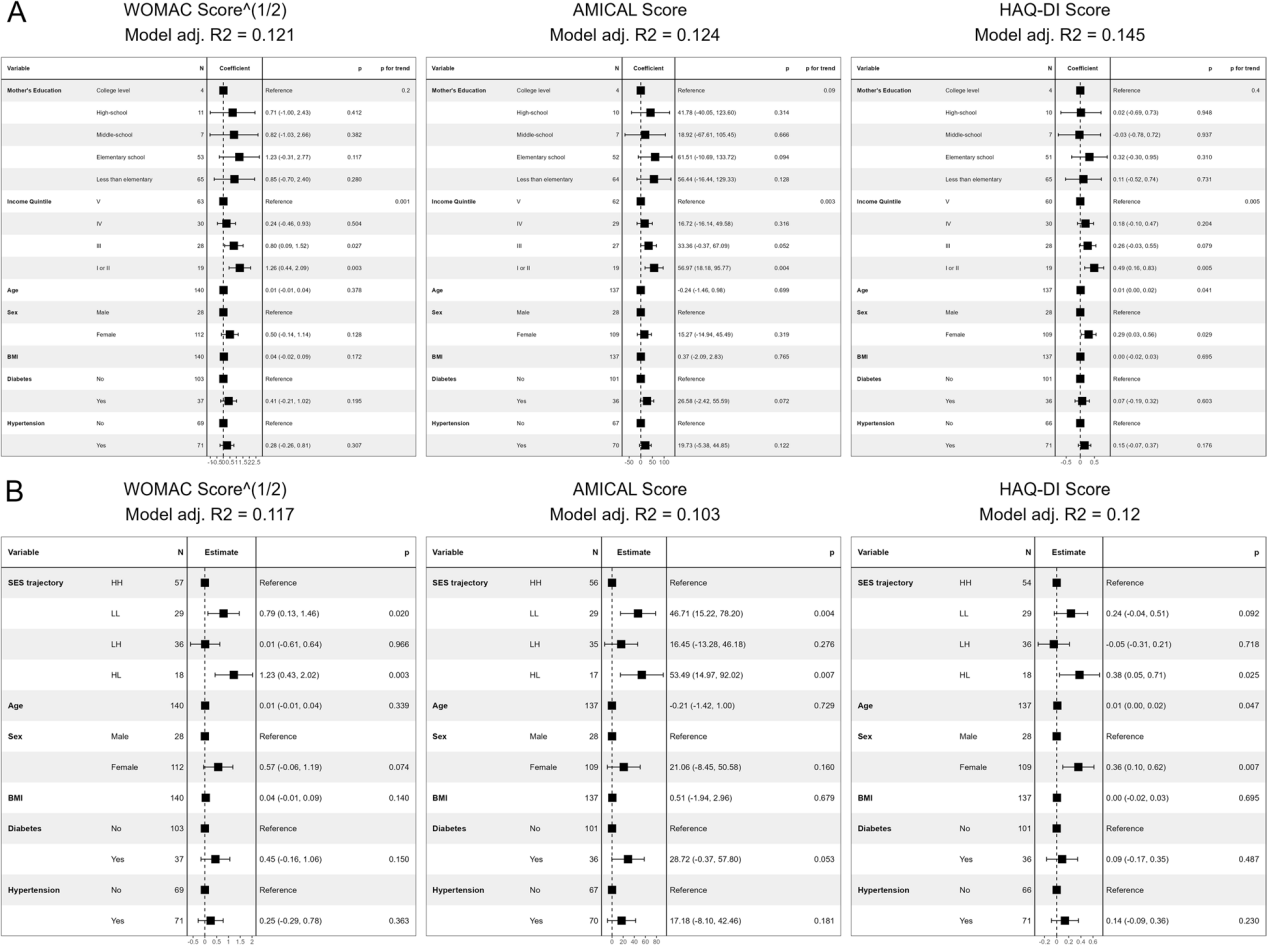
Linear regression models of effect of current and childhood Socioeconomic Status measures on outcome scores **A** Beta coefficients of regression models including both maternal education level and current income level. **B** Beta coefficients of regression models of Socioeconomic Status trajectories. *WOMAC*: Western Ontario and MacMaster Universities Osteoarthritis Index; *AMICAL*: Osteoarthritis of Knee and Hip and Quality of Life; *HAQ-DI*: Stanford Health Assessment Questionnaire Disability Index; *BMI*: Body Mass Index (kg/m^2^). *HH*: High Maternal Education/ High Income; *LL*: Low Maternal Education/ Low Income; *LH*: Low Maternal Education/ High Income; *HL*: High Maternal Education/ Low Income

In mutually adjusted models (i.e. including maternal education and current income in the same model), current income had a significant effect on all outcome scores, while maternal education did not (Fig. 8A). Finally, linear regression models of SES trajectories showed that outcome scores were influenced by these trajectories, although the effect appears to be driven particularly by current income level (Fig. 8B).

## Discussion

Our results show that lower current SES – as measured through income, education or occupation type – is associated with worse functional status, quality of life and disability amongst patients with OA. Our study suggests that in our population, current income is the SES variable that best predicts outcome, as the effect of childhood SES, education and occupation type appears to be largely mediated by it. This can be cautiously interpreted as evidence for the hypothesis that material deprivation pathways may play a role in shaping OA outcome disparities.

Childhood SES also seems to affect some outcomes – particularly quality of life. Although the effect of Childhood SES appears largely mediated by current SES, our study may have been underpowered to detect a direct effect after multiple adjustments.

Our results also provide further evidence that the WOMAC, AMICAL and HAQ-DI scores capture important and similar dimensions – with some variations – of disease severity for hip and knee osteoarthritis in primary care settings. This suggests that the use of these three questionnaires may be equivalent in clinical settings, and that clinicians treating OA patients (particularly in primary care settings) may potentially use any of these questionnaires to assess clinical status in patients with OA, with the choice largely determined by personal preferences and system-level factors. Use of validated tools to assess disease severity can improve management of OA.

As discussed by Luong et al., [8] both socioeconomic behavioral patterns and – more importantly – harmful exposures associated with lower SES through the life course may explain the worse functional status and disability scores, while psychosocial factors may add on to these to influence quality of life [20]. Social-to-biological pathways, such as increased systemic inflammation amongst people of lower SES [35] may well play a role in explaining our results, which are also in line with studies reporting more musculoskeletal pain and generally worse quality of life with decreasing SES.

Important strengths of our study include being, to our knowledge, the first study to examine the relationship between childhood SES and OA outcomes in a Latin American context, and one of few studies to do so worldwide. Major limitations include its cross-sectional nature, small sample size and inherent limitations of measuring a complex construct such as Socioeconomic Status, forcing gross approximations through proxy variables such as income, education and occupation type. Similarly, we were limited by the small number of people in the lowest income groups included in the study, which was due to the fact that our Social Security clinic serves only the families of people with formal employment in a middle to upper-middle class neighborhood.

Taken together with previously mentioned studies showing associations of lower childhood and current SES with higher prevalence and worse outcomes of OA, our study adds to the evidence base on lifecourse social determinants of health as they relate to musculoskeletal diseases. Furthermore, it shows current income to be the most important SES predictor of OA outcomes.

While further research is needed to elucidate the influence of socioeconomic life-course trajectories on both OA incidence and outcomes, and the pathways that mediate this relationship, we believe this study joins others in calling for action on social determinants to reduce health disparities.

## Data Availability

All data generated or analyzed during this study are included in this published article and its supplementary information files.
